# Beyond light signaling: the broad regulatory spectrum of AFPs in plant growth and development

**DOI:** 10.3389/fpls.2026.1930423

**Published:** 2026-09-15

**Authors:** Qizhang Wang, Jiale Yuan, Yongxin Zhang, Bingbing Yang, Fang Lin

**Affiliations:** 1 Ministry of Education Key Laboratory of Cell Activities and Stress Adaptations, School of Life Sciences, Lanzhou University, Lanzhou, China; 2 Gansu Province Key Laboratory of Gene Editing Breeding, School of Life Sciences, Lanzhou University, Lanzhou, China

**Keywords:** ABI5-binding proteins (AFPs), lateral branch, photomorphogenesis, seed germination and dormancy, stress

## Abstract

AFPs (ABI5-BINDING PROTEINS) are a unique class of small, plant-specific proteins, originally identified through their interaction with ABI5 (ABSCISIC ACID INSENSITIVE 5), a pivotal transcription factor in ABA (ABSCISIC ACID) signaling pathway. Emerging evidence indicates that AFPs act as multifunctional integrators governing not only ABA signaling but also a broad spectrum of plant growth, development, biotic and abiotic stress adaptation. This review systematically summarizes the domain architecture, interactome networks, and evolutionary conservation of AFPs, and further highlights the pleiotropic functions of AFPs in seed dormancy and germination, photoperiodic flowering, plant architecture modulation, abiotic stress tolerance and disease resistance. Through diverse molecular mechanisms, including recruitment of corepressors, control of target protein stability, and modulation of chromatin states, AFPs operate as microprotein modulators at critical nodes of phytohormone networks, offering novel insights into the regulatory principles balancing plant growth and stress resilience.

## Introduction

Plant growth and development, as well as adaptation to adverse environments, rely fundamentally on the precise crosstalk and efficient integration of endogenous hormonal signals and external environmental cues. The fine-tuning of signaling transduction pathway is indispensable for maintaining plant life activity homeostasis and adapting to fluctuating environments. ABA serves as a pivotal phytohormone governing plant growth, development, and responses to diverse abiotic stresses (Yoshida et al., 2019). ABI5 belongs to the bZIP (BASIC LEUCINE ZIPPER) family transcription factor and functions as a pivotal regulator in the ABA signaling pathway. Dynamic regulation of its protein stability, transcriptional activation activity, and subcellular localization is essential for the precise control of multiple key physiological processes, including seed dormancy, germination, seedling establishment, flowering, and stress tolerance (Du et al., 2025; Li et al., 2026; Liu et al., 2026; Jinno et al., 2025; Thapa et al., 2026).

AFPs (ABI5-BINDING PROTEINS) represent a group of key regulators that specifically target ABI5 and precisely modulate its molecular functions (Garcia et al., 2008). Early studies simply categorized AFPs as negative regulators of the ABA signaling pathway, with a primary focus on their roles in mediating ABI5 ubiquitination and degradation (Lopez-Molina et al., 2003), as well as relieving ABA mediated repression of seed germination (Lopez-Molina et al., 2001). In recent years, the multifunctional biological properties of AFPs have been progressively uncovered and validated. AFPs not only act as critical molecular switches within the ABA signaling pathway but also function as central hubs that coordinate cross talk among multiple signaling pathways, including those of ABA, GA (GIBBERELLIC ACID), JA (JASMONIC ACID), and light (Finkelstein and Lynch, 2022; Lopez-Molina et al., 2003; Pauwels et al., 2010). Their involvement extends to diverse core biological processes, such as photomorphogenic responses, flowering time control, abiotic stress tolerance, and biotic stress defense. Consequently, AFPs have emerged as important research targets for dissecting the intricate signaling regulatory networks in plants (Vittozzi et al., 2024).

AFPs family members have been gradually identified and characterized in both model plants such as *Arabidopsis thaliana* and a variety of economically important crops, including wheat, cotton, and gladiolus (Ge et al., 2020; Ohnishi et al., 2008; Wu et al., 2016). To date, preliminary insights have been gained into their conserved domain architectures, ABI5-interaction profiles, molecular evolutionary patterns, and certain biological functions (Garcia et al., 2008). In light of these advances, this review focuses on the multifunctional regulatory properties of AFPs. We systematically elaborate on their structural features, interaction mechanisms with ABI5, and evolutionary relationships, providing a comprehensive summary of research progress regarding AFP functions in plant growth and development, abiotic stress tolerance, and disease resistance. Furthermore, we critically discuss the current limitations in this field and outline future research directions and potential agricultural application.

## Discovery of ABI5-binding proteins

Abscisic acid plays essential roles in various growth and developmental processes, including seed dormancy and germination, seedling establishment, stomatal movement, plant morphological architecture establishment, and abiotic stress (Hu et al., 2026; Sun et al., 2025; Teng et al., 2023). ABI5 is the core master regulator of ABA signaling, triggering stress related target genes expression. ABI5 belongs to the bZIP transcription factor which contains multiple conserved regions C1-C5, NC1, NC2, and a C-terminal bZIP domain (**Figure 1B**). Discovered from *Arabidopsis* ABA-insensitive mutant screens, ABI5 mutations abolish ABA responses in seed germination and early seedling development (Tang et al., 2025).

**Figure 1:**
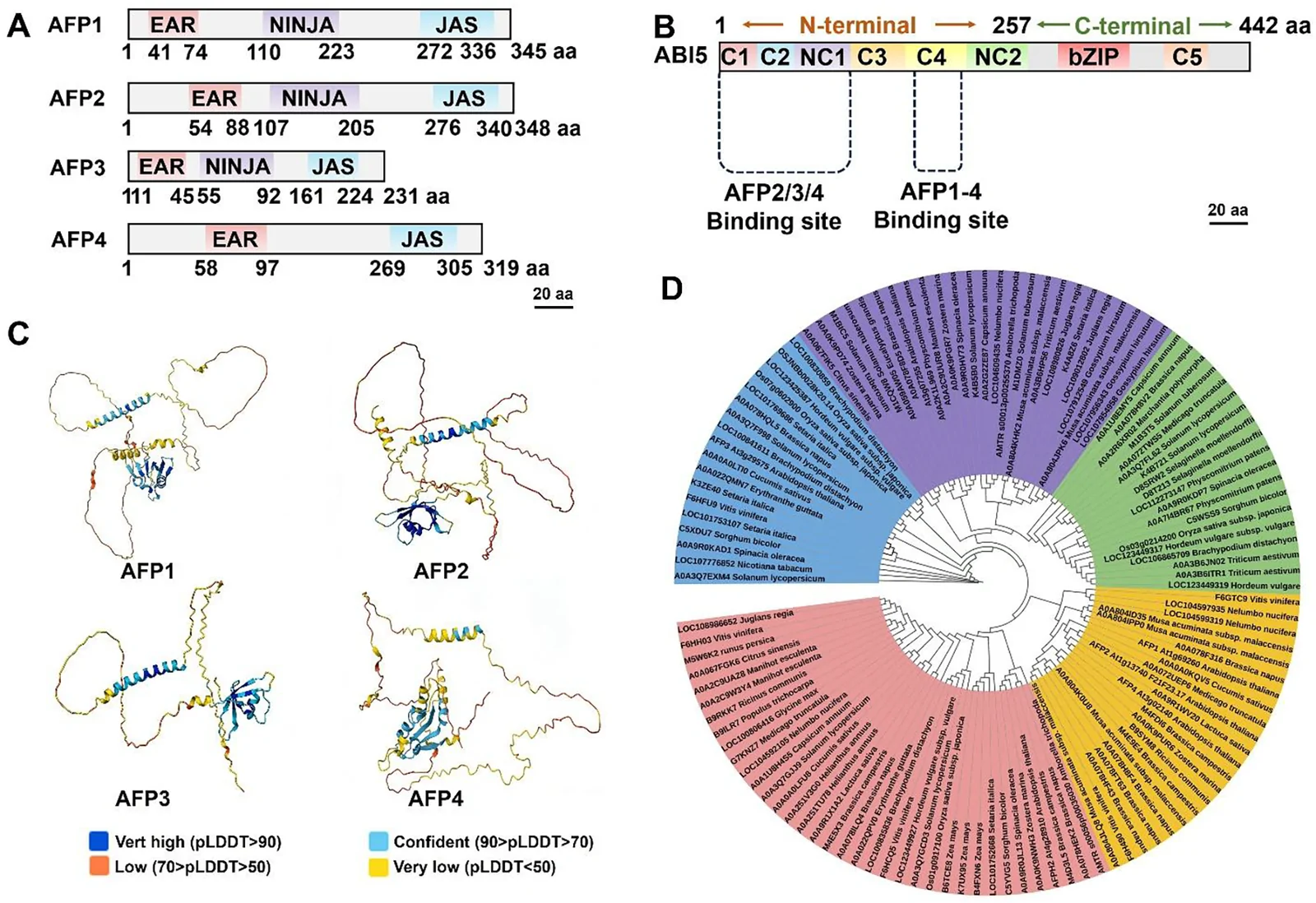
ABI5-binding proteins (AFPs) structural characteristics, interaction patterns and evolutionary distribution. **(A)** Diagram showing the domain structure of AFP1/2/3/4. All AFP proteins contain a conserved N-terminal EAR motif, a central NINJA domain, and a C-terminal JAS domain. Bar=20 aa. **(B)** Diagram showing the domain structure of ABI5 and the binding regions of AFPs. Arabidopsis ABI5 contains multiple conserved domains (C1-C5, NC1, NC2) and a C-terminal bZIP domain. AFP2/3/4 primarily bind the N-terminal region of ABI5 (C1-C3), whereas all AFP members (AFP1-4) associate with the central region (C3-C4). 1-257aa is the N-terminal, while 257-442aa belongs to the C-terminal. Bar=20 aa. **(C)** Predicted 3D structures of Arabidopsis AFP1/2/3/4 were derived from The Arabidopsis Information Resource (https://www.arabidopsis.org). The protein structures were predicted using AlphaFold (Jumper et al., 2021), and the data were retrieved via the UniProt API (https://www.uniprot.org/). Visualization was carried out with PyMol (Sehnal et al., 2021). **(D)** Phylogenetic analysis of the plant AFP/NINJA protein family. The AFP/NINJA family is widely distributed across mosses, gymnosperms, monocots, and dicots. All protein sequence data are derived from the Uniport database (https://www.uniprot.org/).

Upon ABA perception, the phytohormone binds to PYR/PYL (PYRABACTIN RESISTANCE/PYRABACTIN RESISTANCE-LIKE) receptors, which in turn recruit PP2C (PROTEIN PHOSPHATASE 2C) and repress their enzymatic activity (Choi et al., 2000; Uno et al., 2000; Zhou et al., 2026). This derepression allows SnRK2 (SUCROSE NON-FERMENTING 1-RELATED PROTEIN KINASE 2) to phosphorylate ABI5, thereby enhancing both its protein stability and transcriptional activation capacity (Nakashima et al., 2009; Umezawa et al., 2010). Consequently, the expression of late embryogenesis abundant genes, including *EM6* (*EMBRYOGENESIS ABUNDANT PROTEIN 6*), *RAB18* (*RESPONSIVE TO ABA 18*), and other members of the *LEA* (*LATE EMBRYOGENESIS ACCUMULATING*) family, is activated, ultimately inhibiting seed germination and seedling growth (Carles et al., 2002; Fujii et al., 2007). In contrast, when ABA is absent, PP2C proteins associate with SnRK2 to suppress its kinase activity, thus blocking ABI5 activation. Therefore, deciphering the precisely tuned regulatory mechanisms that govern ABI5 activity is of great theoretical importance for understanding how plants balance growth and stress defense. The ABA signaling pathway has been well-characterized in *Arabidopsis thaliana*, and its core regulatory framework is largely conserved among crop species, although several newly-identified regulatory features have been reported in crops. In maize, ZmPYL4 has been documented to enhance plant ABA sensitivity and drought tolerance (Zhong et al., 2025). The full-length transcript *ZmPYL8_T01* also positively modulates drought resistance via ABA dependent signaling (Hao et al., 2026). TaPYL4 elevates ABA sensitivity, which improves water-use efficiency and maintains grain productivity under water-deficit conditions in wheat (Mega et al., 2019). Upon ABA perception, the co-receptor complex activates TaPYR10, which in turn represses TaPP2C30. This repression releases TaSnRK2.10 and subsequently activates TaNF-YC1 (*Triticum aestivum L.* NUCLEAR FACTOR-YC1), conferring improved drought resilience and yield performance (Zhao et al., 2026). In rice, OsNRT1.1B (*Oryza sativa* NITRATE TRANSPORTER 1.1B) has been characterized as a dual receptor for both ABA and nitrate. Under high-nitrogen conditions, OsNRT1.1B preferentially senses nitrate to prioritize nutrient utilization. By contrast, under low-nitrogen and stress conditions, ABA signaling becomes dominant and triggers robust stress-defense responses (Ma et al., 2025).

AFPs were first identified as a novel protein family via yeast two-hybrid screening aimed at isolating ABI5-interacting proteins from germinating Arabidopsis seeds and seedlings (Garcia et al., 2008). The AFP family in *Arabidopsis thaliana* comprises four homologs (AFP1, AFP2, AFP3, and AFP4), with lengths of 345, 348, 231, and 319 amino acids, respectively, and each harbors the canonical EAR (ETHYLENE-RESPONSIVE ELEMENT BINDING FACTOR-ASSOCIATED AMPHIPHILIC REPRESSION), NINJA (NOVEL INTERACTOR OF JAZ), and JAS (JASMONATE ZIM-DOMAIN) motifs, except for AFP4, which carries only the EAR and JAS motifs (**Figure 1A**) (Garcia et al., 2008). Full length of AFPs interact with ABI5 in a member-specific manner, and this interaction is modulated by the phosphorylation status of ABI5. AFP2, AFP3, and AFP4 predominantly bind to the N-terminal segment (C1-C3), whereas all four AFP members are able to associate with the central region (C4), implying that distinct AFP isoforms exhibit selective binding site preferences on ABI5 protein (**Figure 1**) (Lopez-Molina et al., 2003; Wei et al., 2022). Early functional characterization established AFPs as negative regulators of ABA signaling. Transgenic plants overexpressing *AFP1* exhibit ABA insensitive phenotypes, whereas loss-of-function *afp1* mutants display hypersensitivity to ABA. This genetic evidence strongly supports the involvement of AFPs in the ABA signaling cascade. Subsequent studies have considerably broadened the functional landscape of AFPs (Xiao et al., 2021).

The presence of NINJA conserved motif of AFPs points to an evolutionary relationship between the AFP and NINJA families. NINJA represents a plant-specific protein family that has remained highly conserved throughout the evolution of land plants. Proteins belonging to the AFP/NINJA family are widely found in mosses, gymnosperms, monocots, and dicots. In Arabidopsis, AFPs group into a single clade together with their Brassicaceae orthologs, whereas monocot homologs form a distinct, independent clade. These observations demonstrate that the family is evolutionarily conserved during terrestrial plant and has undergone functional divergence in parallel with species differentiation, thereby providing both evolutionary and structural evidence for its critical role as a nexus for crosstalk among multiple phytohormones (**Figures 1C, D**).

AFPs utilize conserved functional domains, including EAR motifs and NINJA-homologous regions, alongside evolutionarily related microproteins, to govern a wide spectrum of biological processes. AFPs exhibit significant molecular homology to the transcriptional co-repressor NINJA (Pauwels et al., 2010). Similar to NINJA, several AFP isoforms physically associate with the co-repressor family members TOPLESS (TPL) and TPL-related proteins (TPRs). The interaction spectrum and binding strength between AFPs and TPL/TPR co-repressors vary drastically across distinct AFP isoforms: AFP2 and AFP3 display robust physical interactions with TPL and almost all TPR paralogs, whereas AFP1 only weakly binds a small subset of these co-repressors, and AFP4 lacks detectable interactions with any TPL/TPR proteins (Lynch et al., 2017). Microproteins such as LNJ (LITTLE NOVEL INTERACTOR OF JAZ) are small single-domain polypeptides sharing sequence homology to the multi-domain protein AFP2 which also contains the NINJA domain. Microproteins tune the biological activity of target proteins through two primary modes: assembling alternative protein complexes or sequestering key signal transduction components. LITTLE NINJA, a NINJA type microprotein, promotes JA (JASMONIC ACID) signaling likely by alleviating the assembly of JAZ-NINJA-TPL repressor complexes (Hong et al., 2020). It has been demonstrated that overexpression of full-length *AFP2* causes a delay in flowering, whereas the *afp2* mutant exhibits earlier flowering than wild-type controls. These phenotypes are in contrast to those observed in transgenic *LNJ-OX* plants, which display dwarfism and loss of apical dominance. This suggests that the dominant-negative function of LNJ differs from the biological functions of AFP2.

## The role of AFPs in light signaling and photoperiodic flowering

Plants employ an extensive set of photoreceptors, including phytochromes, cryptochromes, phototropins, and UVR8 (UV RESISTANCE LOCUS 8), to sense the diverse wavelengths of sunlight. These light inputs are then transduced into intracellular signals that precisely govern plant growth and development (Liu et al., 2020). In Arabidopsis, light signaling is tightly controlled by multiple photoreceptors and their downstream components. In darkness, COP1 (CONSTITUTIVELY PHOTOMORPHOGENIC 1) associates with SPA (SUPPRESSOR OF PHYTOCHROME A) proteins to form an active E3 ubiquitin ligase complex, that targets photomorphogenesis-promoting factors, such as HY5 (ELONGATED HYPOCOTYL 5) and BBX21 (B-BOX DOMAIN PROTEIN 21)/STH2 (SALT TOLERANCE HOMOLOG 2), for ubiquitination and degradation, thereby suppressing photomorphogenesis. Meanwhile, PIFs (PHYTOCHROME-INTERACTING FACTORS), members of the bHLH (BASIC HELIX-LOOP-HELIX) transcription factor family, function as positive regulators of skotomorphogenesis by regulating the expression of a wide range of genes involved in cell elongation (Willige et al., 2021). Upon light exposure, activated photoreceptors trigger the dissociation of the COP1-SPA complex and degradation of PIFs, thereby releasing the repression and initiating both photomorphogenic development and flowering program (Zhu et al., 2020; Wang et al., 2021). Subcellular localization studies have shown that coexpression of AFP2 and ABI5 leads to their co-localization in nuclear bodies, where COP1 is also present. Given that COP1 serves as a central switch of photomorphogenesis and possesses E3 ubiquitin ligase activity, it has been proposed that AFP2 may function as an adaptor protein that recruits ABI5 or other substrates to COP1-containing nuclear bodies for degradation. This working model not only provides a subcellular basis for elucidating AFP2-mediated protein turnover but also hints at a potential function for AFP2 in the regulation of photomorphogenesis (Lopez-Molina et al., 2003).

Flowering time, a critical developmental transition for plant adaption to seasonal environmental changes, is governed by the convergence of multiple regulatory cues, among which photoperiod, temperature, and phytohormones are prominent (Luo et al., 2025; Verrico and Preston, 2025). The photoperiodic flowering pathway comprises three fundamental components, namely, light signal input via photoreceptors, the circadian clock oscillator, and a downstream set of flowering-related regulatory genes (Shim et al., 2017). AFP2 has been shown to serve as a key integrator of photoperiodic flowering, thereby fine-tuning plant reproductive development. In the photoperiodic flowering pathway, AFP2 exhibits a circadian expression pattern that peaks during the night, and it mediates transcriptional repression through a multi-domain interaction mechanism. Specially, its C terminal with JAS domain binds to the central flowering regulator CO (CONSTANS/BBX1), while the N terminal conserved EAR motif recruits the transcriptional corepressor TPR2 (Topless-related protein 2), resulting in the formation of a CO-AFP2-TPR2 ternary complex. This complex exerts two distinct but coordinated effects. It promotes ubiquitin-dependent degradation of CO protein during the night, reducing CO accumulation, and it also recruits histone deacetylases via TPR2 to remodel the chromatin at the major floral integrator gene *FT* (*FLOWERING LOCUS T*), repressing *FT* transcription and ultimately negatively regulating flowering under long day conditions. Notably, this flowering regulatory module operates independently of the ABA-FLC (FLOWERING LOCUS C) pathway. Genetic evidence confirms that *AFP2* overexpression reduces transcript levels of *FT* and its downstream flowering targets, causing a marked delay in flowering, whereas the *afp2* mutant displays enhanced CO protein stability and early flowering (Chang et al., 2019). Furthermore, J3 (DNAJ HOMOLOG 3) specifically interacts with AFP2 and antagonizes the association between AFP2 and CO. This interaction attenuates AFP2 mediated CO degradation, stabilizing CO protein abundance, which in turn activates *FT* expression and positively regulates photoperiodic flowering. Moreover, genetic complementation assays have demonstrated that J3 completely rescues the late flowering phenotype induced by *AFP2* overexpression, indicating that J3 and AFP2 function antagonistically to fine-tune flowering time under photoperiod control (Zhi et al., 2025) (**Figure 2**).

**Figure 2:**
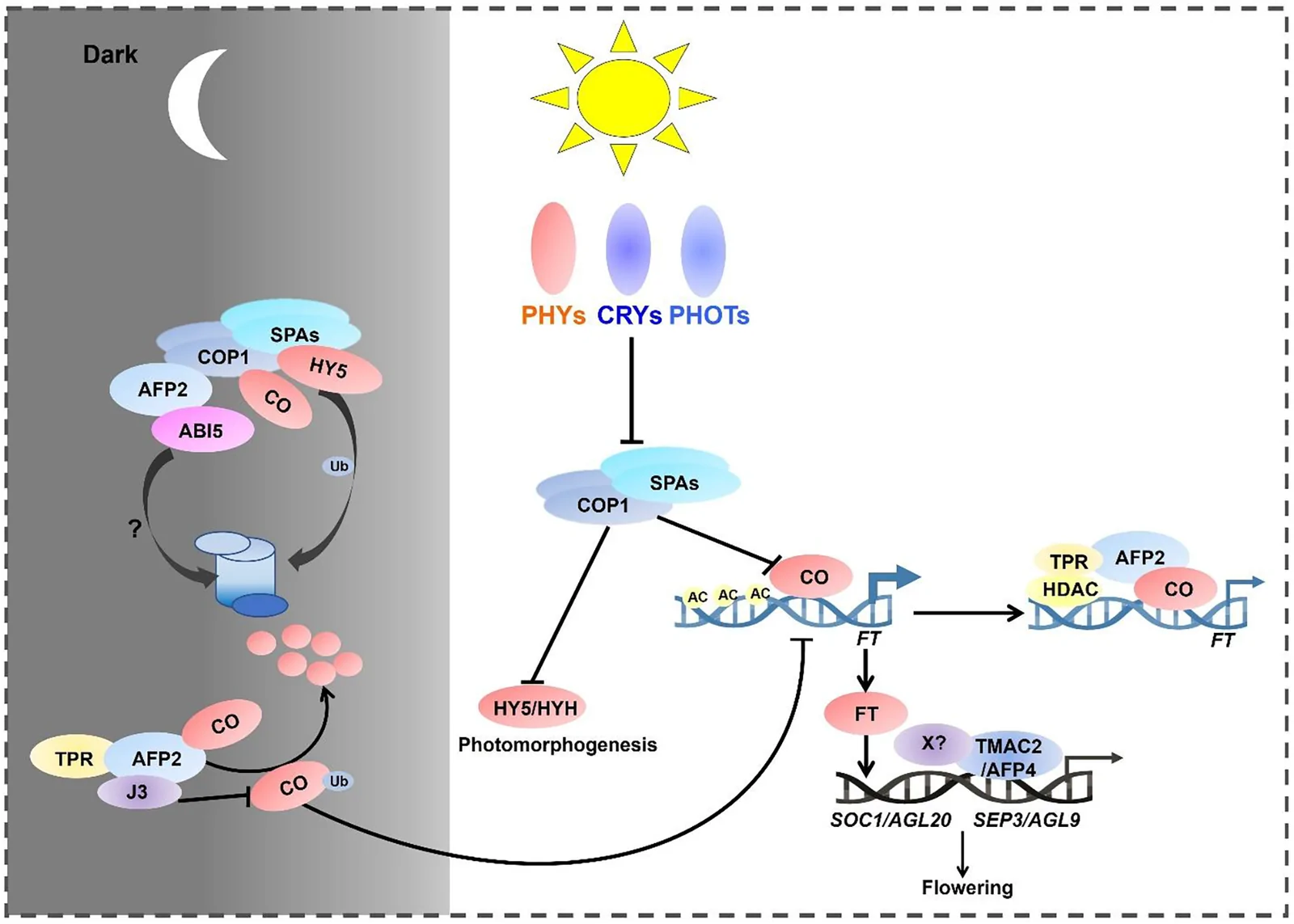
AFP proteins mediate light signaling to coordinate flowering. AFP4/TMAC2 negatively regulates flowering by repressing the floral integrator *SOC1/AGL20*. In the photoperiodic flowering pathway, circadian *FT* expression is controlled by AFP2 via a light-dark switch. In the light, J3 counteracts AFP2, leading to CO accumulation and *FT* induction; in the dark, however, AFP2 assembles with CO and TPR2/HDAC into a repressor module that triggers CO turnover and reduces histone acetylation to suppress *FT*.

At the transcriptional level, overexpression of *TMAC2* (*AFP4*) suppresses the transcript levels of *SEP3/AGL9* (*SEPALLATA 3*), *SOC1/AGL20* (*SUPPRESSOR OF OVEREXPRESSION OF CONSTANS 1*), *SEX1* (*STARCH EXCESS* 1), alongside genes involved in starch breakdown, implying a pleiotropic function in floral transition and carbohydrate catabolism (Huang and Wu, 2007). AFP4 is thought to act analogously to AFP2, potentially through association with CO or alternative factors, but the underlying details await further investigation. Collectively, AFPs serve as critical node that binds J3, CO, and TPR2, contributing to control photoperiod flowering (**Figure 2**).

## AFPs regulate seed germination and dormancy

AFPs are a family of core repressors within the plant ABA signaling cascade. Through their capacity to coordinate interplays among signaling components, epigenetic marks, various post-translational modifications, and hormone crosstalk, AFPs exert precise control over seed dormancy, germination, secondary dormancy, organ dormancy, and tolerance to abiotic stress. In ripened seeds during primary seed dormancy, WRKY36 (WRKY DNA-BINDING PROTEIN 36) occupies the W-box motif within the *DOG1* (*DELAY OF GERMINATION 1*) promoter. This is followed by WRKY36-mediated recruitment of AFP2, which occurs through physical interaction with the NINJA and JAS domains of AFP2. AFP2 then engages the co-repressor TPR2, leading to the assembly of a ternary WRKY36-AFP2-TPR2 complex at the *DOG1* promoter. Subsequently, TPR2 recruits histone deacetylases (HDACs, HISTONE DEACETYLASE), which catalyze histone deacetylation at the *DOG1* locus. This epigenetic modulation compacts chromatin architecture and represses *DOG1* transcription, ultimately breaking primary seed dormancy (Deng et al., 2023). ABA and GA operate antagonistically to balance seed development. DELLA proteins, which serve as repressors of GA signaling, potentiate ABA responses and promote the establishment of deep seed dormancy. In contrast, both AFP1 and AFP2 act as DELLA antagonists. Notably, their overexpression in the DELLA hyperaccumulating mutant *sleepy1* mitigates DELLA mediated phenotypes, conferring a substantial reduction in storage protein deposition, alongside a marked reversal both hyper dormancy and ABA hypersensitivity. Through this interplay, AFPs act as balancing hub that integrates ABA and GA signals, effectively dampening their antagonistic effects and ensuring proper developmental progression (Finkelstein and Lynch, 2022). AFPs are involved in multiple genetically independent routes that control ABI5 degradation within the protein turnover network. Specifically, AFP1 associates with the E3 ligase PUB35 (PLANT U-BOX 35) to ubiquitinate ABI5 and target it for 26S proteasome degradation, which promotes seed germination and seedling growth. However, phosphorylation of ABI5 at T201 and T206 disrupts its interaction with AFP1 and impairs its binding to PUB35, attenuating PUB35 mediated degradation *in vivo* and consequently causing the ABI5 accumulation (Du et al., 2024). Interestingly, overexpression of *AFP2* in the *dwa1 dwa2* double mutant yields both strong ABA resistance and improved dehydration tolerance, even though ABI5 remained abundant in seed populations with up to 90% germination. These results suggest that the AFP-regulated seed ABA sensitivity and dehydration tolerance are not mediated by ABI5 loss but rather may involve distinct regulatory mechanisms pathways governing these two stress-adaptation properties (Lynch et al., 2022) (**Figure 3**).

**Figure 3:**
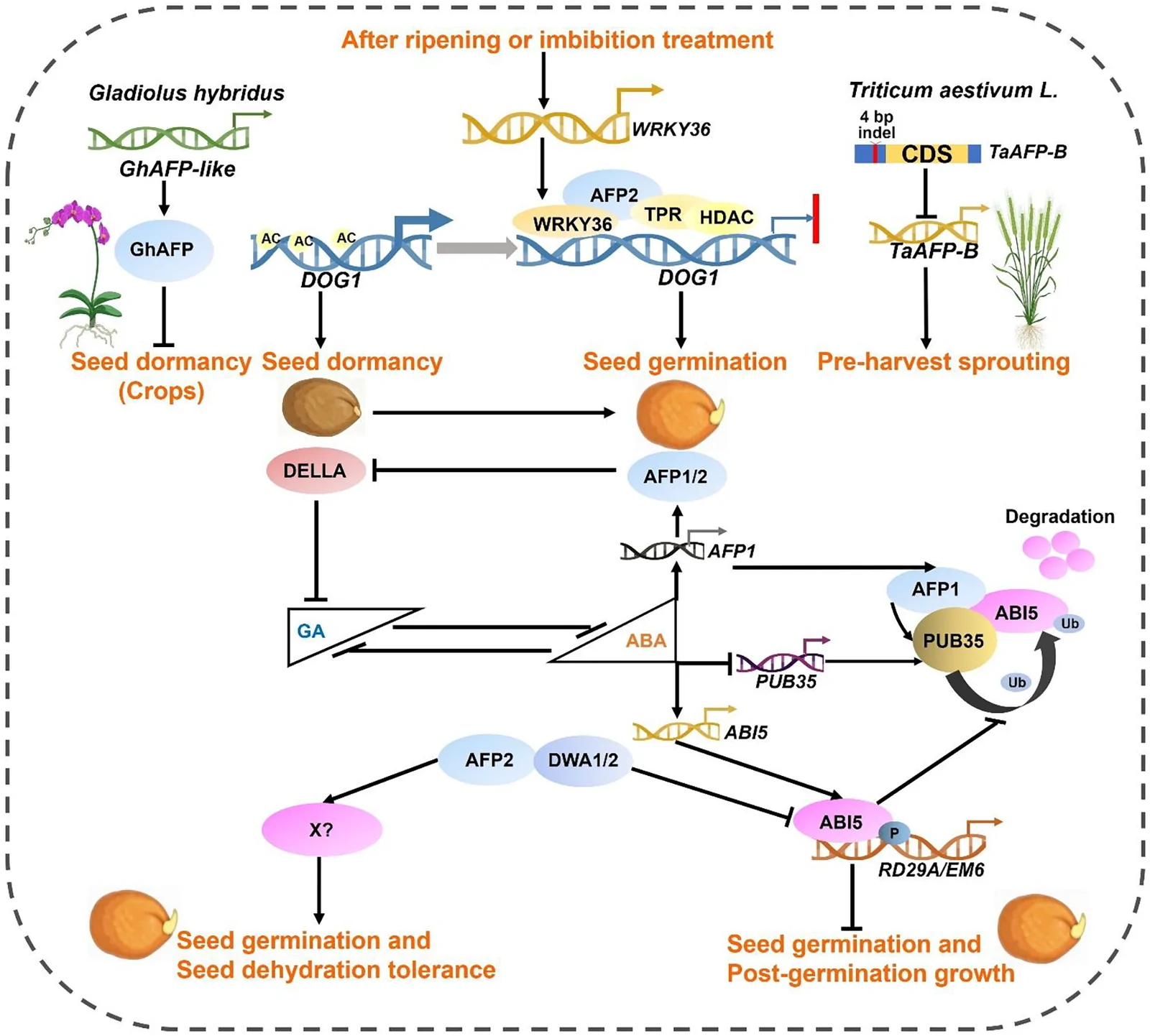
AFP family proteins regulate seed dormancy and germination. AFPs balance ABA-GA crosstalk by antagonizing DELLA proteins. AFP2 independently regulates ABA sensitivity and dehydration tolerance. It also represses ABI5-SOM to relieve heat-induced secondary dormancy. AFP2-WRKY36-TPR2 complex represses *DOG1* to release primary dormancy. AFP1 collaborates with PUB35 to mediate ABI5 degradation, promoting seedling growth. Natural variations in TaAFP-B and conserved GhAFP-like function indicate broad conservation in crops and horticultural plants. This figure was created with BioRender (https://www.biorender.com).

The functional conservation of AFP proteins extends well beyond model plants to crops and horticultural species. In wheat (*Triticum aestivum* L.), TaAFP-B locus harbors multiple natural allelic variants, with inDel polymorphisms in its promoter and 5’ untranslated region (UTR) exerting profound effects on gene transcription, mRNA stability, and translation efficiency. These molecular variations, in turn, modulate endogenous ABA content and tissue sensitivity to ABA, thereby determining the strength of seed dormancy and the degree of resistance to pre-harvest sprouting. As such, *TaAFP-B* emerge as a valuable functional marker for the genetic improvement of sprouting tolerance in wheat breeding (Feng et al., 2019, 2022). In the horticultural crop *gladiolus*, a newly identified nucleus-localized protein, GhAFP-like (*Gladiolus hybridus*), has been shown to negatively regulates the ABA pathway, controls corm dormancy, and mediates auxin responses, expanding our knowledge of AFPs in organ-specific dormancy and phytohormone interplay (Wu et al., 2016) (**Figure 3**). Taken together, AFP family proteins govern seed dormancy, germination, secondary dormancy, and organ dormancy through intricate multilayered molecular framework, encompassing transcriptional regulation, epigenetic modifications, ubiquitination, and phosphorylation.

## Plant architecture modulation

Plant architecture encompassing height and lateral branch or tiller development represents a critical agronomic trait that directly influences both crop yield potential and cultivation adaptability. AFP family proteins and their derived microproteins play essential regulatory roles in shaping plant architecture through the modulation of hormone signaling cascades. In Arabidopsis, modification of the AFP2 gene, which harbors a NINJA domain, generates a class of LNJ (LITTLE NINJA) microprotein mutant. These mutants display a dwarf phenotype reminiscent of JA signaling mutant, and their transcriptomic profiles overlap substantially, indicating that AFP2 derived LNJ-like microproteins regulate plant height via modulation JA signaling. In grasses, the LNJ microprotein acts as a dominant rheostat of JA signaling. Overexpression of *LNJ* profoundly remodels plant architecture. In short statured grasses, elevated *LNJ* expression inhibits stem elongation while promoting lateral branching and tillering. This regulatory effect is transferable across species; ectopic expression of *LNJ* in barley and rice likewise leads to dwarfism and enhanced tillering, providing novel molecular targets and strategies for architecture improvement in cereal crops (Hong et al., 2020). In cucumber (*Cucumis sativus* L.), CsAFP3-like, an AFP protein with a conserved ABI5-binding domain, is closely associated with lateral branch development. Its transcription is drastically attenuated in the lateral branch deficient *mbl* (*mutant-branchless*) mutant, which concomitantly exhibits excessive ABA accumulation in leaf axils. VIGS (Virus induced gene silencing) assays confirmed that CsAFP3-like positively regulates ABA mediated inhibition of lateral branch development. These findings establish a crucial role for the AFP family in regulating lateral branch development in dicot plants (Yang et al., 2025) (**Figure 4**). Collectively, AFP family proteins, whether deployed in their full-length form or as derived microprotein isoforms, orchestrate plant architectural traits across both monocot and dicot species. Their regulatory influence is channeled primarily through JA signaling, which modulates stature and branching, and ABA signaling, which governs lateral outgrowth, thereby laying both a theoretical groundwork and a practical blueprint for molecular breeding strategies designed to tailor plant architecture for enhanced crop performance.

**Figure 4:**
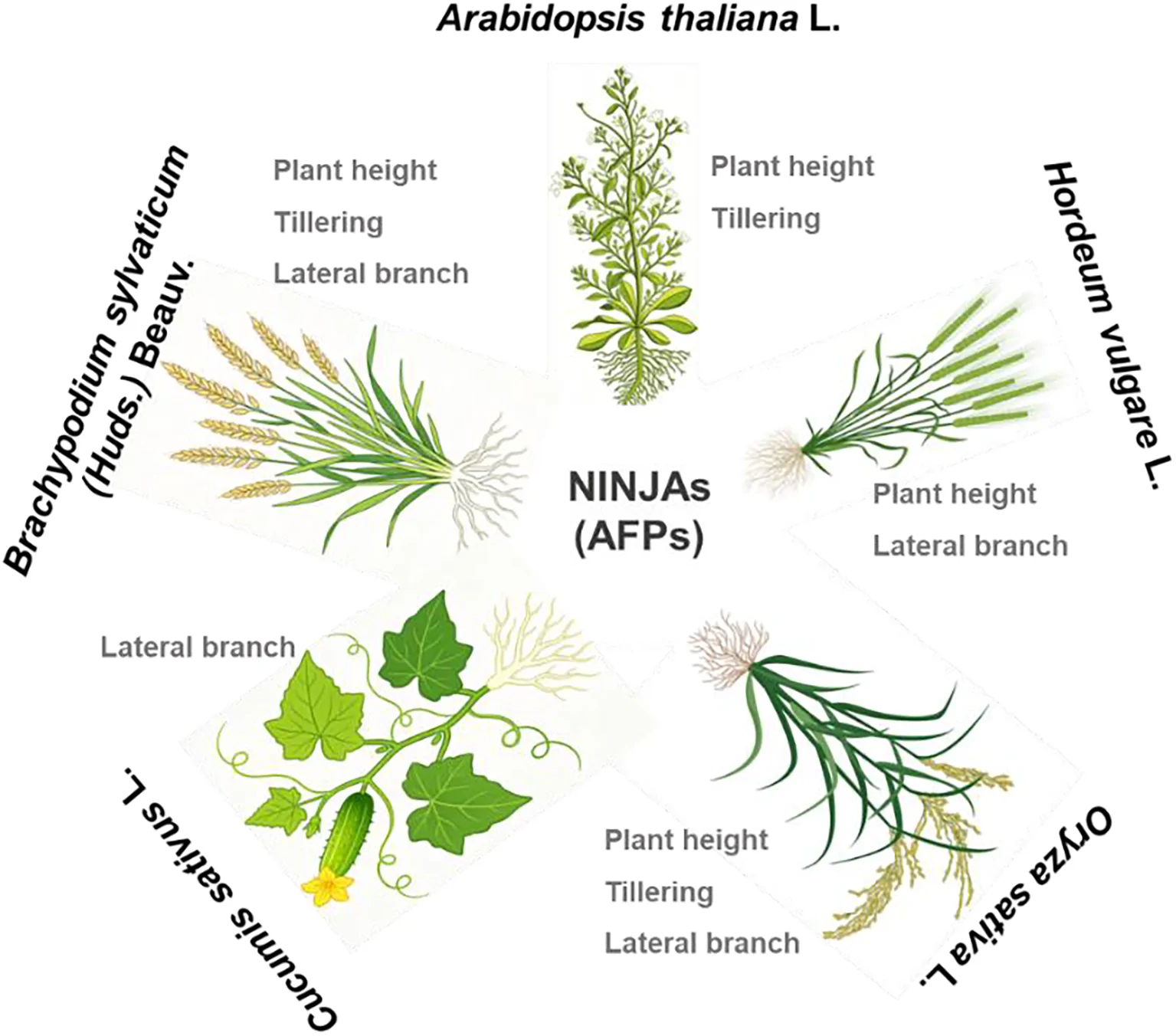
NINJA/AFP family proteins regulate plant architecture across diverse species. The NINJA/AFP protein family plays a pivotal role in shaping plant architecture, determining traits such as height, tiller number, and lateral branch formation. This regulatory function is conserved across a broad spectrum of species, including *Arabidopsis thaliana L., Hordeum vulgare L.*, *Oryza sativa L.*, *Cucumis sativus L.*, and *Brachypodium sylvaticum (Huds.) Beauv*. This figure was created with BioRender (https://www.biorender.com).

## AFPs control plant responses to abiotic stress

As critical repressors of ABA signaling, AFPs participate broadly in multiple abiotic stress responses, including salt, osmotic, drought, high temperature, and oxidative stresses. In Arabidopsis, AFP members are induced by ABA, dehydration, high salinity, and other stress signals. Accordingly, loss-of-function mutants of *AFP1* and *AFP2* exhibit hypersensitivity to osmotic stress, and both genes act upstream of *ABI5*. Under salt stress, the impaired germination or seedling vigor observed in these mutants correlates with elevated ABI5 protein levels relative to wild-type controls, although it remains unclear whether this accumulation represents the primary cause of growth inhibition or a secondary consequence thereof. Notably, while both *ABI5* and *AFP* genes are transcriptionally upregulated by ABA, the ABI5 to AFP ratio increases under high ABA concentrations, thereby maintaining growth arrest under severe stress (Garcia et al., 2008). AFP1 also collaborates with the E3 ubiquitin ligase PUB35 to ubiquitinate ABI5 and drive its 26S proteasome-mediated degradation. Moreover, ABI5 phosphorylation impairs its complex stability with AFP1, allowing fine-tuning of ABA and salt stress responses (Du et al., 2024). In addition, AFP2 directly interacts with the core salt stress kinase SOS2 (SALT OVERLY SENSITIVE 2). Through this association, AFP2 stability is enhanced and ABI5 turnover is accelerated, effectively lifting the suppression of seed germination under salt stress (Wang et al., 2024). AFP4, also known as TMAC2 (TWO OR MORE ABRES CONTAINING GENE 2), is involved in promoting shorter primary roots and maintaining Na^+^ homeostasis in response to salinity. Overexpression of *TMAC2* led to a marked increase in *HKT1* (*HIGH-AFFINITY K^+^ TRANSPORTER 1*) transcripts, reaching even higher levels than those observed in the Col-0 *UAS_GAL4_::HKT1* background line. Interestingly, when *TMAC2* and *HKT1* were co-expressed, the expression of *ABI4* and *ABI5* was upregulated. However, overexpression of *TMAC2* alone did not affect the transcript abundance of either *ABI4* or *ABI5*. Collectively, these observations point to the existence of a transcriptional feedback between *HKT1* and *TMAC2* (Alshareef et al., 2025) (**Figure 5**).

**Figure 5:**
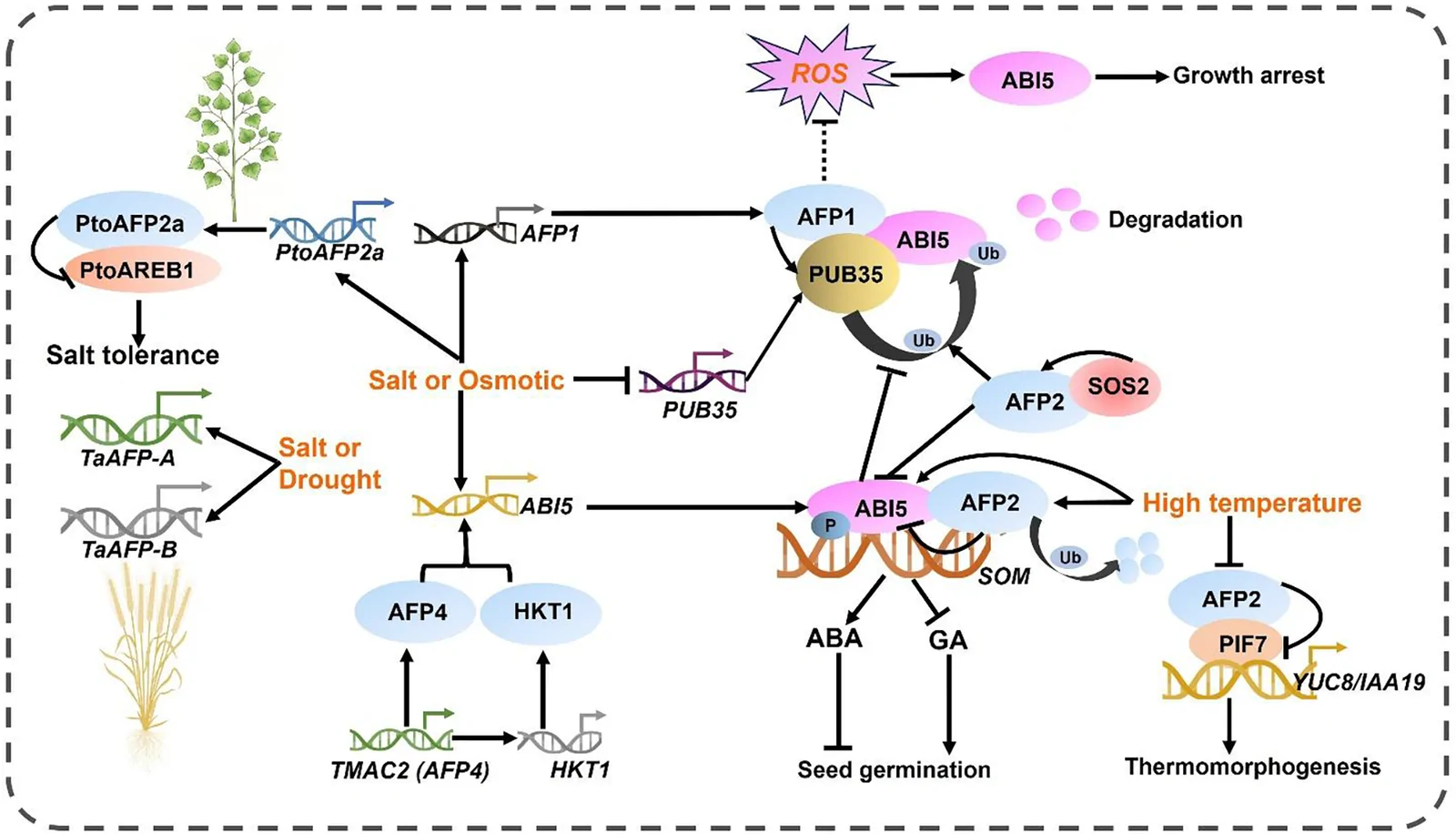
Role of AFP proteins in regulating plant abiotic stress. Under salt stress, AFP1 interacts with PUB35 to mediate ABI5 degradation and fine-tune ABA signaling. SOS2 stabilizes AFP2, which in turn facilitates ABI5 degradation and relieves salt-induced germination arrest. AFP4 (TMAC2) modulates HKT1 to maintain Na^+^ homeostasis. In poplar, PtoAFP2a interacts with PtoAREB1 to negatively regulate salt tolerance. Under high temperature, AFP2 associate with ABI5 to activate *SOM*, disturb ABA/GA balance, and induce secondary seed dormancy. With thermomorphogenesis, AFP2 interacts with PIF7 to inhibit auxin biosynthetic genes such as *YUC8*, while high temperature promotes AFP2 degradation to release PIF7 repression and induce hypocotyl elongation. Drought induces Wheat *AFP* genes expression. In addition, AFP1 is essential for preserving ROS homeostasis and regulating oxidative stress defense.

Meanwhile, full length of AFP2 physically interacts with PIF7, a core component of the photomorphogenic cascade, to modulate thermomorphogenic responses. Elevated ambient temperatures trigger the proteolysis of AFP2, consequently abolishing its inhibitory effect on PIF7. In contrast, at normal temperature, AFP2 directly engages with PIF7, effectively dampening its transactivation of auxin biosynthetic genes such as *YUC8 (YUCCA 8*) and *IAA19* (*INDOLE-3-ACETIC ACID INDUCIBLE 19*). This interaction ultimately restrains hypocotyl elongation and acts as a negative regulator for thermomorphogenic growth (Deng et al., 2021). In addition, high temperature induces secondary dormancy in seeds. AFP2 functions in a ABI5-dependent manner to suppress the transcription of *SOM* (*SOMNUS*), a pivotal negative regulator of seed germination, while simultaneously upregulating GA biosynthetic genes and downregulating ABA biosynthetic genes. This coordinate transcriptional reprogramming reshapes the endogenous hormone balance, effectively alleviating the high temperature-induced secondary dormancy and thereby facilitating seed germination under heat stress (Chang et al., 2018).

To uncover novel genetic determinants within the ROS (REACTIVE OXYGEN SPECIES) regulatory network, a forward genetic screen was performed on approximately 2000 randomly selected Arabidopsis T-DNA insertion lines. Seedlings were grown on agar medium supplemented with either the superoxide-eliciting herbicide methyl viologen or the catalase inhibitor 3-amino-triazole, enabling the identification of mutants with altered ROS sensitivity or resistance. This unbiased approach led to the identification of *AFP1* as a bona fide component of ROS homeostasis, with its disruption significantly attenuating oxidative resilience (Petrov et al., 2013). In woody plants, the poplar *PtoAFP2a* (*Populus tomentosa* Carr.) is markedly induced by salt stress and ABA treatment. This protein interacts with the transcriptional activator PtoAREB1s (*Populus tomentosa* Carr. ABA-RESPONSIVE ELEMENT BINDING PROTEIN 1) and represses its activity, thereby establishing a negative feedback loop within ABA signaling that ultimately attenuates salt tolerance in poplar (Zhou et al., 2025). Similarly, the wheat *TaAFPs* family members are also implicated in stress adaptation. Both *TaAFP-A* and *TaAFP-B* respond to salt and drought cues. Intriguingly, *TaAFPs* display a considerably broader organ-specific expression profile relative to their Arabidopsis orthologues, hinting that monocot AFPs have undergone functional diversification to meet the demands of stress adaptation (Ohnishi et al., 2008). Collectively, the AFP family acts as a central hub linking ABA signaling to multiple abiotic stress signals. Through both evolutionary conserved and lineage-specific molecular strategies, AFPs orchestrate plant acclimation to salinity, drought, osmotic, heat, and oxidative stresses, serving as a key regulatory module that balances stress defense and growth development in plants.

## AFPs facilitate plant disease resistance

NINJA is a pivotal factor in JA signaling cascade that governs plant immunity. The bioactive conjugate JA-Ile (Jasmonoyl-L-isoleucine) orchestrates both defense responses and development programs through activation of the transcription factor MYC2 (MYC-RELATED TRANSCRIPTION FACTOR 2). In the absence of active signaling, JAZ repressors engage NINJA, which in turn recruits TPL/TPR co-repressors via its conserved EAR motif, thereby assembling a transcriptional repression complex that curtails MYC2 activity and holds JA signaling in a quiescent state. The stability of the NINJA-TPL module is critical for JA homeostasis, and its dysfunction improves plant disease resistance (Pauwels et al., 2010). Systematic phylogenetic analyses have traced AFPs and NINJA proteins to a common evolutionary origin, as evidenced by their substantial sequence homology. This ancestral relationship not only points to a functional linkage between the two families but also provides compelling evolutionary support for the involvement of AFPs in NINJA mediated JA signaling, laying a foundation for exploring their cooperative roles in plant disease resistance. In rice (*Oryza sativa* L.), activation of antiviral immunity hinges on the targeted degradation of NINJA family components. Upon recognition of viral coat proteins, the E3 ubiquitin ligase OsRBRL (*Oryza sativa* RING1-IBR-RING2-TYPE UBIQUITIN LIGASE) ubiquitinates and degrades OsNINJA3, an AFP homologous adaptor, which relieves JA pathway repression and triggers antiviral defense. This regulatory cascade represents a conserved mechanism for virus perception and defense activation in rice (Huang et al., 2025). A separate but analogous route involves OsBBI1 (*Oryza sativa* BROAD-SPECTRUM BLAST RESISTANCE 1), another E3 ligase, which ubiquitinates and degrades several JA co-repressors, notably OsNINJA1 and OsJAZ6/7/8, thus alleviating their suppression of resistance to both rice blast and bacterial blight. OsNINJA1 functions primarily by dampening pathogen-induced ROS accumulation and constraining immune activation. OsBBI1 and OsJAZ6 interact with OsNINJA1, while OsJAZ6 interacts with Myelocytomatosis protein 2 (OsMYC2) and OsMYC3, and *OsOXO4* (*Oryza sativa* OXALATE OXIDASE) and *OsZIP9* (*Oryza sativa ZRT-IRT-LIKE PROTEIN 9*) were identified as direct targets of OsMYC2. Moreover, OsMYC2 forms homodimers and heterodimers with OsMYC3, which plays a synergistic role in OsMYC2-mediated activation of target gene transcription (Bi et al., 2025) (**Figure 6**). Collectively, these two distinct NINJA degradation pathways constitute the core regulatory network underlying broad-spectrum disease resistance in rice. Studies in cotton further validate the intimate link between the AFP family and NINJA mediated JA signaling, consistent with their documented evolutionary homology. GhAFP8, a prominent member of the cotton AFP family, is transcriptionally induced by JA, ABA, and SA signals. Overexpression of *GhAFP8* significantly enhances resistance to *Verticillium* wilt, whereas its silencing severely compromises disease resistance (Zhou et al., 2024) (**Figure 6**). Given the evolutionary homology between AFPs and NINJA, pathogen-triggered turnover of NINJA family proteins is controlled by E3 ubiquitin ligases (OsRBRL and OsBBI1), meanwhile NINJA dependent transcriptional repression, along with other associated processes, is tightly coordinated with the functional interplay of the AFP protein family. These components are organized into a hierarchical regulatory network that constitutes a conserved molecular framework underpinning plant defense against both viral and fungal pathogens. This integrated system not only provides critical theoretical support and valuable gene resources for breeding disease-resistance crops.

**Figure 6:**
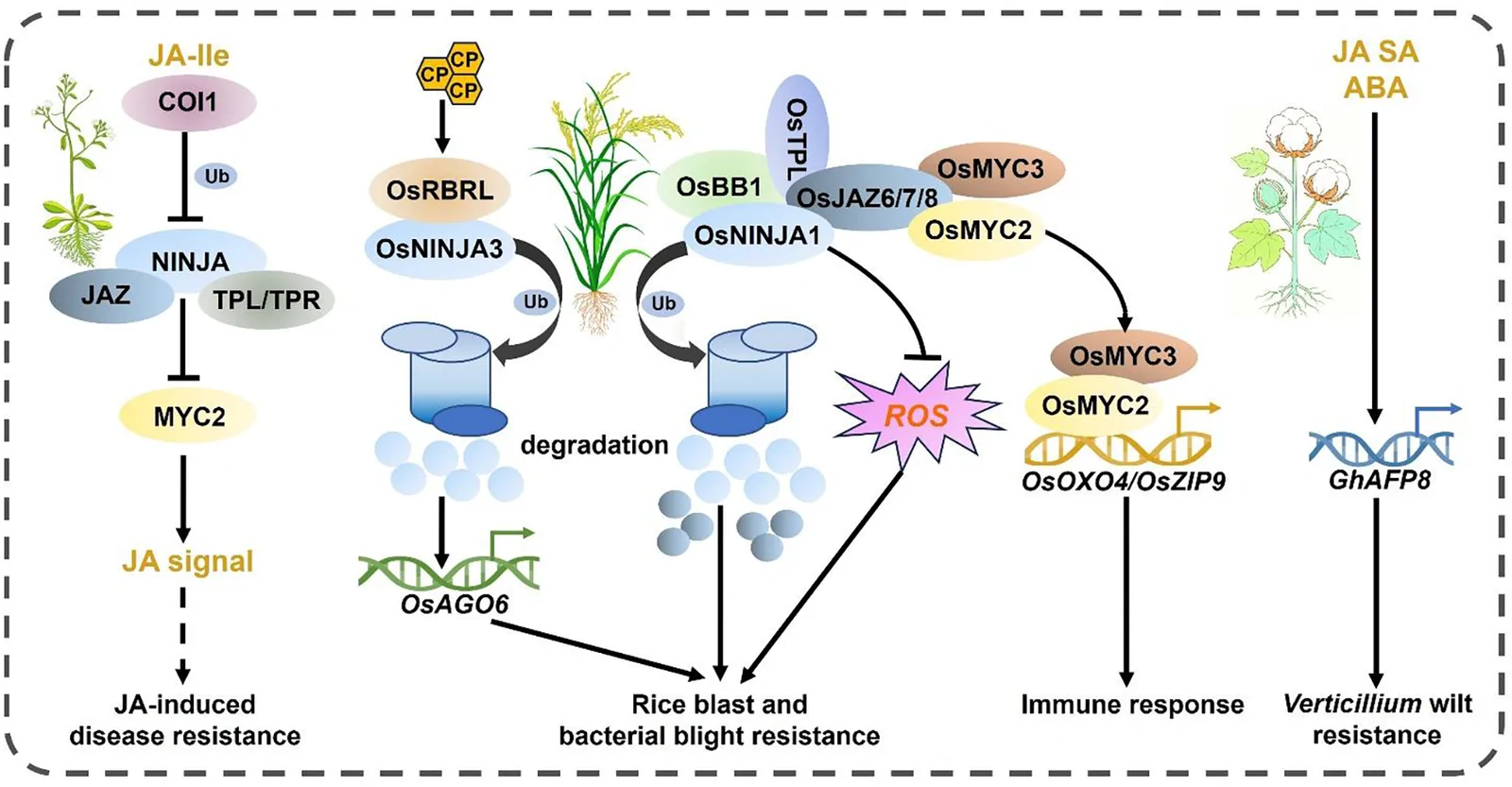
AFP/NINJA proteins bridge disease resistance with hormonal signaling networks. In the absence of JA-Ile, JAZ repressors associate with NINJA, which in turn recruits TPL/TPR co-repressors to form a transcriptional repression complex, thereby repressing MYC2-dependent transcriptional activity. Upon recognition of the viral coat protein (CP) in rice, the E3 ligase RBRL degrades OsNINJA3 to derepress JA signaling, which in turn upregulates antiviral genes including *OsAGO6*. OsBBI1 degrades OsNINJA1 and OsJAZ6/7/8 to release OsMYC2/3, triggering immune responses. OsNINJA1 also functions by dampening pathogen-triggered reactive oxygen species (ROS) accumulation and constraining immune activation. *GhAFP8*, a cotton gene induced by ABA, JA, and SA, functions to activate hormone‐related and defense pathways. Consequently, this activation strengthens plant immunity against *Verticillium dahliae*. This figure was created with BioRender (https://www.biorender.com).

## Comprehensive outlook and future research directions

AFPs are a family of small molecule proteins unique to plants, serving as a core regulatory hub that integrates ABA, GA, JA, auxin, and environmental cues to regulate plant growth, development, and stress adaptation. AFP1 accelerates PUB35 mediated ABI5 ubiquitination and proteasomal degradation to fine-tune ABA responses, leading to seed germination and seedling establishment. AFP2 forms ternary complexes with WRKY36 and TPR2 to epigenetically silence the master dormancy gene *DOG1* histone deacetylation. Concurrently, AFP2 integrates ABA and GA signals through physical interaction with DELLA proteins, balancing dormancy imposition and germination. Under high temperature, AFP2-ABI5 modules suppress *SOM* expression while reprogramming GA/ABA biosynthesis genes, alleviating secondary dormancy. Moreover, AFP2 functions as a circadian regulated molecular hub that integrates light signals into flowering time control. It forms a CO-AFP2-TPR2 ternary complex that simultaneously promotes CO protein degradation and represses *FT* transcription via chromatin remodeling. This module operates independently of the ABA-FLC pathway and is antagonized by J3, providing intricate photoperiodic timing. Furthermore, full length AFPs and their derived microproteins (e.g., LNJ from AFP2) regulate plant height, tillering, and lateral branching through JA signaling (modulating stature and branching) and ABA signaling (governing lateral bud outgrowth). This regulatory influence is conserved across monocots and dicots, offering valuable targets for crop architecture improvement.

AFPs also broadly participate in plant responses to salt, osmotic, drought, heat, and oxidative stresses through multiple distinct mechanisms. These include the SOS2 mediated stabilization of AFP2 and accelerated turnover of ABI5 under saline conditions, temperature dependent modulation of PIF7 activity that fine-tunes thermomorphogenic growth, negative feedback loops. PtoAFP2a-PtoAREB1s module in poplar, that prevent excessive stress signaling, and the maintenance of ROS homeostasis. Based on their evolutionary homology with the NINJA family, AFPs are recruited into JA signaling to defend against pathogen attack. In cotton, GhAFP8 enhances resistance to *Verticillium* wilt by competing with NINJA for binding to TPL/TPR corepressors. In rice, pathogen triggered degradation of NINJA family components, such as OsNINJA3 by the E3 ligase OsRBRL and OsNINJA1 by OsBBI1, represents a conserved defense module that confers both antiviral and antifungal immunity.

Although significant progress has been made in AFPs research, several fundamental questions remain unresolved. AFP1-AFP4 exhibit distinct ABI5-binding preferences, tissue expression patterns, and downstream targets, yet the structural basis (beyond domain architecture) for these differences remains unclear. High resolution 3D structures and dynamic conformational analyses of each member are urgently needed. AFPs are known to function in multiple hormonal and environmental signaling pathways, including those of ABA, GA, JA, auxin, light, and temperature. However, the mechanisms underlying their signal specific switching among ABA, GA, JA, light, and temperature responses remain largely elusive. It is still unclear whether this switching involve conformational changes, competitive binding among interaction partners, or post-translational modifications that modulate partner selectivity. To detect hormone- or stimulus-induced conformational changes in AFPs, hydrogen–deuterium exchange mass spectrometry (HDX-MS), Forster resonance energy transfer (FRET) with fluorophore-tagged AFPs, or intrinsic tryptophan fluorescence spectroscopy can be employed. These methods enable monitoring of structural dynamics in response to different signaling inputs. Additionally, nuclear magnetic resonance (NMR) or small-angle X-ray scattering (SAXS) can provide high-resolution conformational information under varying ligand or cofactor conditions. To evaluate competitive binding among interaction partners (e.g., ABI5, other transcription factors, or regulatory proteins), co-immunoprecipitation (Co-IP) and pull-down assays should be performed using protein extracts from plants treated with each hormone (ABA, GA, JA, light, or temperature shifts). Quantitative binding affinities can be measured by surface plasmon resonance (SPR) or isothermal titration calorimetry (ITC) in the presence of competing partners or signaling molecules. Furthermore, fluorescence polarization or microscale thermophoresis (MST) can be used to assess binding competition in a high-throughput manner. To distinguish direct competition from allosteric effects, peptide arrays or domain-swapping mutants can be tested. To investigate the role of PTMs (Post-translational modification) in partner selectivity, a comprehensive phosphoproteomic and ubiquitinomic analysis of AFPs under each signaling condition should be conducted using liquid chromatography tandem mass spectrometry (LC-MS/MS). Site-specific mutants (e.g., phosphor null, phosphor mimetic, or ubiquitination-defective variants) can then be generated and tested for their interaction profiles with known partners via yeast two-hybrid (Y2H) or split-luciferase complementation assays. In parallel, *in vitro* kinase/phosphatase assays with recombinant AFPs and candidate enzymes can reveal whether specific PTMs directly alter binding selectivity. To integrate these molecular findings with cellular dynamics, live-cell imaging of fluorescently tagged AFPs should be performed to monitor subcellular relocalization upon hormone treatments, while bimolecular fluorescence complementation (BiFC) or proximity ligation assays can visualize *in situ* interactions with specific partners under each condition. Combining these experimental strategies, and cross-validating results with genetic mutants in corresponding signaling pathways. It will enable a systematic dissection of whether conformational shifts, competitive binding, PTMs, or their interplay underlie the signal-specific switching of AFPs.

Beyond ABI5, AFPs target PIF7, CO, WRKY36, and DELLA. What sequence or structural features define substrate recognition? Is there a conserved “phosphor-code” or degron motif that enables selective targeting of diverse transcription factors? Are AFPs phosphorylated by SnRK2, MAPKs (MAPK KINASE KINASE), or other kinases? Elucidating how these modifications affect AFP stability, interaction affinity, subcellular dynamics, and the reciprocal regulation of ABI5 phosphorylation is essential for advancing our knowledge of the molecular basis of AFPs-mediated regulation.

During critical developmental phase transitions, AFPs serve as key coordinators that balance growth promotion with stress adaptation via multiple mechanistic routes. On the one hand, AFP-directed ABI5 turnover and AFP-DELLA mediated suppression of the ABA/GA ratio collectively favor germination. On the other hand, lower ABA/GA ratio reduces ABA sensitivity and decreases storage protein deposition, which may counteract the beneficial effects of germination. Under elevated temperatures, AFPs drive germination even at the cost of growth inhibition. Yet, the mechanistic basis by which AFPs interpret and prioritize conflicting signals to achieve an optimal balance remains to be elucidated. While TaAFP-B InDel polymorphisms are linked to pre-harvest sprouting resistance in wheat, the functional alleles of AFPs in rice, maize, cotton, and other staple crops remain largely unexplored. The fact that AFPs are negative regulators of ABA signaling, while initially counterintuitive, is precisely their greatest asset for engineering climate-resilient crops. Rather than a blunt “more ABA is equivalent to better survival” approach, AFPs offer rheostatic control over stress sensitivity, enabling crops to avoid the severe growth penalties typically associated with constitutive ABA activation. Here is how this knowledge can be translated into practical, field-deployable strategies:

### Climate responsive flowering time and harvest index optimization

The discovery of the CO-AFP2-TPR2 ternary complex, which operates independently of the ABA-FLC pathway and is antagonized by J3, provides a precise molecular lever for photoperiodic tuning. As day-length patterns shift with climate change, AFP2 variants that modulate the stability of CO and the chromatin remodeling of *FT* could be engineered to adjust flowering windows without disrupting vernalization requirements. Moreover, the existence of full-length AFPs and their derived microproteins (e.g., LNJ from AFP2) that regulate plant height, tillering, and lateral branching-conserved across monocots and dicots-offers a goldmine for ideotype breeding. By using CRISPR-Cas9 to knock in microprotein-specific translation initiation sites or to alter the processing of these small peptides, breeders can independently modify branching architecture (via JA signaling) and bud outgrowth (via ABA signaling) to suit high-density planting systems, without compromising stress resilience.

### Engineering multi-stress tolerance via spatiotemporal “stress brake” modules

SOS2-mediated stabilization of AFP2 under salinity, or the temperature-dependent AFP2-ABI5-SOM module that alleviates secondary dormancy under heat provide allele-specific targets for fine-tuning stress responses. For example, breeders could edit the promoter regions of AFP2 so that its expression is induced specifically under combined heat with salinity conditions, thereby uncoupling thermal escape from osmotic damage. In staple cereals, CRISPR-mediated base editing could introduce phospho-mimetic or phospho-null mutations at residues targeted by SOS2, effectively creating a “salt-dial” that adjusts ABI5 turnover rates specifically under saline irrigation, without altering basal ABA responses during normal growth. Given the preliminary indications linking AFPs to ROS signaling, several approaches can be employed to uncover the underlying molecular mechanisms. Multiple independent *afp* loss-of-function alleles (and complemented lines) could be used to rigorously reproduce the oxidative stress phenotypes (e.g., methyl viologen and 3-amino-triazole sensitivity/resistance). Real-time ROS accumulation (H_2_O_2_, O_2_·^-^) can be measured with fluorescent probes (e.g., H_2_DCFDA, DHE), while antioxidant enzyme activities (SOD, CAT, APX, GPX) are to be quantified in both untreated and stressed plants. Reporter lines carrying *AFP1* promoter-GUS or promoter-GFP constructs should be generated to map tissue-specific and stress-inducible expression. Monitoring of *AFP1* transcript and protein levels under different ROS-inducing conditions (time course and dose response) can be achieved through qRT-PCR and immunoblotting. Screening for physical interactors of AFP1 may be performed using yeast two hybrid (Y2H) or immunoprecipitation-mass spectrometry (IP-MS). RNA-seq analysis comparing wild type and *afp1* mutants, with and without ROS stress, is designed to identify differentially expressed genes, particularly those implicated in ROS scavenging and signaling (e.g., MAPKs, calcium, hormones). Since ROS often modulate protein function via oxidation, phosphorylation, or ubiquitination, examination of AFP1 post-translational modifications under stress conditions by anti-phospho and anti-ubiquitin immunoblotting or LC-MS/MS is warranted. Candidate modification sites, such as cysteines for redox-sensing and ser/thr residues for phosphorylation, can be mutated to assess their impact on AFP1 activity and stress responses. Epistatic relationships can be tested by crossing *afp1* with mutants of well-known ROS regulators, including *rbohD/F*, *cat2*, *oxi1*, *mpk3/6*. Evaluation of growth, yield, and seed set under long-term mild oxidative stress (e.g., low doses of MV or natural light stress) would link molecular findings to whole-plant fitness. Through the combination of genetics, transcriptomics, and biochemistry, it becomes possible to advance from initial observation toward a mechanistic understanding of AFP1-mediated ROS integration, which ultimately contributes to oxidative resilience in crops.

### Broad-spectrum stress resistance through JA-TPL/TPR competition

The evolutionary homology with NINJA and the JA defense module opens an unprecedented opportunity for dual-purpose resistance. Since GhAFP8 enhances *Verticillium* wilt resistance by competing with NINJA for TPL/TPR corepressors, and since pathogen-triggered degradation of OsNINJA1/3 confers antiviral and antifungal immunity in rice, AFPs can be strategically overexpressed or stabilized to prime active the JA pathway. A practical pipeline would involve screening natural AFP alleles in major crops for their intrinsic binding affinity to TPL/TPR versus NINJA. By introducing gain-of-competition variants (e.g., AFP alleles with enhanced TPL-binding domains), we could generate crops with built-in broad-spectrum resistance to soil-borne fungi and viral pathogens, simultaneously reducing reliance on chemical fungicides while preserving yield potential. Current evidence focuses on viral and fungal pathogens. Do AFPs also regulate bacterial disease, herbivory, or symbiotic interactions? Additionally, roles in heavy metal toxicity, hypoxia, nutrient deficiency, and combined stress scenarios remain virtually unexplored. It will be possible to drive AFP expression under tissue-specific or stress-inducible promoters to achieve “stress-responsive defense and unstressed normal growth” phenotypes.

### Exploiting the “counterintuitive” negative regulator logic for precision breeding

The most powerful translational strategy arises directly from the counterintuitive nature of AFP function. Since AFPs attenuate ABA responses, traditional overexpression screens are inappropriate. Instead, the focus should be on quantitative fine-tuning rather than loss- or gain-of-function extremes. Integrating GWAS (Genome-Wide Association Study) with pan-genomic structural variants across rice, maize, and cotton populations can identify natural InDels (akin to TaAFP-B in wheat) that alter AFP protein stability or interaction surfaces without abolishing its regulatory logic. These “hypomorphic” or “hyper-stable” alleles can then be introgressed via marker-assisted selection. Rather than knocking out AFPs (which would cause hyper-sensitivity and growth arrest), targeted prime editing can modify the specific amino acid residues involved in PUB35-mediated ubiquitination (for AFP1), DELLA-binding (for AFP2), or TPL/TPR recruitment (for AFP2 and GhAFP8). This would allow us to shift the equilibrium-for instance, reducing AFP-ABI5 affinity just enough to delay germination during unseasonal warm spells (preventing pre-harvest sprouting), while retaining sufficient AFP activity to avoid stunting. The ternary complex structures (e.g., AFP2-DELLA-TPR2) represent druggable interfaces. High-throughput screening for natural or synthetic compounds that transiently stabilize the AFP2-DELLA interaction could serve as exogenous sprays-applied just before predicted drought events-to transiently tilt the ABA/GA ratio in favor of water conservation, without permanently altering the crop’s genetic makeup.

Collectively, the true larger ramification is that AFPs serve as cellular “conflict-resolution” nodes, interpreting simultaneous inputs from light quality, developmental age, abiotic stress and biotic attack. The future of AFP research lies not in viewing them solely as ABA-signaling terminators, but as customizable homeostatic dials that link stress perception to developmental output. By embracing their negative-regulatory, context-dependent, and multi-pathway nature, we can move beyond the “single-gene resistance” paradigm toward a holistic “stress-resilient architecture” approach-ultimately delivering crops that are shorter, tiller-optimized, disease-tolerant, and climate-adaptable, all while maintaining robust yields under escalating environmental volatility. Such efforts will not only deepen our understanding of plant adaptability and developmental plasticity but also provide crucial theoretical foundations and genetic resources for breeding high yield, multi-stress tolerant, and climate resilient crops.
